# Pulmonary hypertension at admission predicts ICU mortality in elderly critically ill with severe COVID-19 pneumonia: retrospective cohort study

**DOI:** 10.1186/s12947-023-00300-0

**Published:** 2023-01-18

**Authors:** Marko Kurnik, Helena Božič, Anže Vindišar, Petra Kolar, Matej Podbregar

**Affiliations:** 1 Department of Internal Intensive Medicine, General Hospital Celje, Oblakova ulica 5, 3000 Celje, Slovenia; 2 Faculty of Medicine, University of Ljubljana, Ljubljana, Slovenia

**Keywords:** COVID-19, Elderly, ICU, Mortality, Echocardiography, Lung ultrasound, Pulmonary artery systolic pressure, Pulmonary hypertension

## Abstract

**Background:**

Point-of-care ultrasound (POCUS) is a useful diagnostic tool for non-invasive assessment of critically ill patients. Mortality of elderly patients with COVID-19 pneumonia is high and there is still scarcity of definitive predictors. Aim of our study was to assess the prediction value of combined lung and heart POCUS data on mortality of elderly critically ill patients with severe COVID-19 pneumonia.

**Methods:**

This was a retrospective observational study. Data of patients older than 70 years, with severe COVID-19 pneumonia admitted to mixed 25-bed, level 3, intensive care unit (ICU) was analyzed retrospectively. POCUS was performed at admission; our parameters of interest were pulmonary artery systolic pressure (PASP) and presence of diffuse B-line pattern (B-pattern) on lung ultrasound.

**Results:**

Between October 2020 and March 2021, 117 patients aged 70 years or more (average age 77 ± 5 years) were included. Average length of ICU stay was 10.7 ± 8.9 days. High-flow oxygenation, non-invasive ventilation and invasive mechanical ventilation were at some point used to support 36/117 (31%), 39/117 (33%) and 75/117 (64%) patients respectively. ICU mortality was 50.9%. ICU stay was shorter in survivors (8.8 ± 8.3 vs 12.6 ± 9.3 days, *p* = 0.02). PASP was lower in ICU survivors (32.5 ± 9.8 vs. 40.4 ± 14.3 mmHg, *p* = 0.024). B-pattern was more often detected in non-survivors (35/59 (59%) vs. 19/58 (33%), *p* = 0.005). PASP and B-pattern at admission, and also mechanical ventilation and development of VAP, were univariate predictors of mortality. PASP at admission was an independent predictor of ICU (OR 1.061, 95%CI 1.003–1.124, *p* = 0.039) and hospital (OR 1.073, 95%CI 1.003–1.146, *p* = 0.039) mortality.

**Conclusions:**

Pulmonary artery systolic pressure at admission is an independent predictor of ICU and hospital mortality of elderly patients with severe COVID-19 pneumonia.

## Background

Coronavirus disease 2019 (COVID-19) is a multi-system disease caused by the severe acute respiratory syndrome coronavirus 2 (SARS-CoV-2) [1].

In-hospital mortality of older hospitalised COVID-19 patients is high [2]. Frailty is independently associated with higher in-hospital mortality, even though COVID-19 patients with frailty are presented to the hospital earlier and with less severe symptoms [2]. Factors associated with mortality of elderly people diagnosed with SARS-CoV-2 who lived in institutions or who were hospitalized because of the disease were dementia, diabetes, chronic kidney disease and hypertension [3].

Echocardiography has become a useful clinical tool both in medical wards and in critical care setting, since it is able to provide information on concomitant clinical conditions (e.g., heart failure) and on current hemodynamic status and heart–lung interactions [4]. In COVID-19 patients, the echocardiographic assessment of the right ventricle (RV) represents a pivotal element in the understanding of current disease status and in monitoring disease progression [5].

Most patients hospitalized with COVID-19 had lung ultrasound abnormalities on admission. Point-of-care ultrasound (POCUS) can aid in risk stratification for patients with COVID-19 admitted to general wards and into ICUs [6].

Aim of current study was to evaluate predictive value on mortality of point-of-care echocardiography and lung ultrasound (LUS) in elderly critically ill at ICU admission.

## Methods

### Setting

A retrospective study was conducted in mixed 25-bed, level 3, ICU in General and Teaching Hospital Celje, Slovenia during a 6-month period (from October 2020 to March 2021).

### Patients

This ICU was specially dedicated for treatment of SARS-CoV-2 positive adult (≥ 18 years old) patients. The diagnosis of COVID-19 was made in the presence of at least one positive real-time polymerase chain reaction (RT-PCR) test for SARS-CoV-2 on respiratory specimen/s (nasopharyngeal swab, sputum, and/or lower respiratory tract specimens).

Pregnant women were not treated in our ICU. Only elderly critically ill patients (≥ 70 years old) were included into final analysis of the study. Patients with previous history of systolic left (severely diminished left ventricular ejection fraction, < 30%) or right heart failure and/or previously detected pulmonary hypertension (estimated PASP > 35 mmHg) were not included in the study.

Study was conducted after receiving positive agreement from the Republic of Slovenia National Medical Ethics Committee (No. 0120–168/2021/7, 22 July 2021) and from the Institutional Review Board of General Hospital Celje (No. 17/KS/2021–1, 5 March 2021). Informed consent was omitted due to the retrospective nature of the study.

### Patient data

The following patient data was collected from the hospital electronic database BIRPIS21 (SRC Infonet, Kranj, Slovenia): basic demographic data, previous medical history, chronic illnesses (e.g., malignant disease, arterial hypertension, diabetes, heart failure, chronic obstructive pulmonary disease, chronic renal disease).

### Laboratory

Majority of laboratory analysis was done in General Laboratory of our institution. In laboratory data we focused on admission data and also on clinically worst laboratory data during the whole ICU stay (e.g., lowest pH, highest pCO_2_, lowest pO_2_, highest D-dimer, highest troponin T, highest procalcitonin (PCT), highest C-reactive protein (CRP), highest creatinine, highest leucocyte count, etc.).

### Echocardiography

Transthoracic echocardiographic exam with cardiac probe (GE Vivid S60 Ultrasound machine, GE healthcare, USA) was performed by the intensive care specialist at admission. Protocol based admission echocardiography data was recorded in the intensive care information system (Centricity Critical Care, GE healthcare, USA). The examination was recorded for later off-line re-evaluation on the workstation (GE EchoPAC Clinical Workstation Software, GE Healthcare, USA) by the experienced ICU care specialist focusing more on cardiology (MP). The following data was collected: left ventricular ejection fraction (LVEF) by eyeballing, velocity time integral (VTI) in left ventricular outflow tract (LVOT), tricuspid annular plane systolic excursion (TAPSE), minimal and maximal inferior vena cava diameter (VCI min, VCI max, respectively) and pulmonary artery systolic pressure (PASP). PASP was estimated with Bernoulli equation from maximal velocity of tricuspid regurgitation and central venous pressure (CVP) [7]. CVP was measured invasively. The definitions of RV dysfunction were based on the American Heart Association Guidelines [8].

### Lung ultrasound

Lung ultrasound exam with a linear probe (GE Vivid S60 Ultrasound machine, GE Healthcare, USA) was performed by the intensive care specialist at admission. Eight lung areas [9] were examined, four on each side (apical anterior area around upper midclavicular line, basal anterior area around lower midclavicular line, apical lateral area around upper midaxillary line and basal lateral area around lower midaxillary line). Patients were classified into: A-pattern when A lines were detected in all examined positions, mixed pattern, when A and B lines were detected, or diffuse B-lines pattern, when only B-lines or lung consolidations were detected in all examined positions. Presence of pleural effusions and lung consolidations were also recorded [10]. The protocol-based admission LUS data was collected from the intensive care information system (Centricity Critical Care, GE Healthcare, USA). Please refer to Study limitations section for additional comments.

### Treatment

Treatment data was collected from the intensive care information system (Centricity Critical Care, GE healthcare, USA). All patients were initially treated with methylprednisolone 1 mg/kg body weight daily as adapted from protocol used at the time [11].

For characterizing respiratory support, we collected the following data: performance of self proning and proning during mechanical ventilation; frequency and duration of high-flow nasal cannula (HFNC) oxygen therapy, non-invasive ventilation (NIV) and invasive mechanical ventilation (IMV). Duration of HFNC and NIV ventilation was guided by using ROX index [12]. In patients supported with IMV the data of maximal positive end-expiratory pressure (PEEP), maximal peak pressure and maximal tidal volume ever recorded during treatment was recorded. Use of nitric oxide inhalation therapy, norepinephrine, levosimendan, systemic thrombolytic treatment and renal replacement therapy were also recorded.

### Complications and mortality

The intensive care and hospital information system were used to collect data on complications (e.g., ventilator associated pneumonia (VAP), catheter-related bloodstream infections, urosepsis, fungal infections), ICU and hospital mortality.

#### Definitions

VAP was defined as new or changing chest X-ray infiltrate/s occurring more than 48 h after initiation of invasive mechanical ventilation, plus both of the following: (i) new onset of fever (body temperature ≥ 38 °C)/hypothermia (body temperature ≤ 35 °C) and/or leukocytosis (total peripheral white blood cell count ≥ 10,000 cells/µL)/leukopenia (total WBC count ≤ 4500 cells/µL)/ > 15% immature neutrophils; (ii) new onset of suctioned respiratory secretions and/or need for acute ventilator support system changes to enhance oxygenation [13].

Catheter-related bloodstream infection (CRBSI) was defined as the presence of bacteremia originating from an intravenous catheter. Microbiological samples were performed using BacT/ALERT SA (aerobic) and BacT/ALERT SN (anaerobic) bottles incubated in the BacT/Alert 3D blood culture instrument (bioMérieux, Ballerup, Denmark) [14].

Fungal infection was diagnosed as previously described [15].

### Primary outcome

Primary outcomes were mortality at ICU discharge and at hospital discharge. The relationship of echocardiography and lung ultrasound data at admission to the primary outcomes.

### Secondary outcome

Secondary outcome was an exploration of the relationship between compilations and ICU mortality.

### Sample size estimation

To achieve 80% power with type-I error rate of 0.05 (two-tailed) for detecting statistically significant differences in PASP between ICU survivors and non-survivors, a sample size of 53 subjects per group (total of 106 patients) was determined to be required. PASP difference of 8 mmHg with standard deviation of 14 mmHg between ICU survivors and non-survivors was assumed. MedCalc ver. 12.5 (MedCalc Software Ltd, Ostend, Belgium) was used for sample size estimation.

### Statistical analysis

Data were summarized as mean (± standard deviation) for metric variables; absolute and relative frequencies for categorical variables. Tests for normal distributions were carried out on continuous variables. The Student's *t*-test was used for metric variables and Chi-Square for categorical data. Univariate and multivariate logistic regression modelling, with odds ratio calculations, was used to test the relationship between echocardiography, LUS data, complications and ICU/hospital mortality. A receiver operating characteristic (ROC) curve analysis was used for testing the predictive ability of PASP for ICU/hospital mortality. The analyses were performed using SPSS v.25.0 software package (SPSS Inc., Chicago, IL, USA) and MedCalc ver. 12.5 (MedCalc Software Ltd, Ostend, Belgium). A *p*-value of < 0.05 was considered to define statistical significance.

## Results

Three-hundred-forty-three patients were admitted into our ICU during the study period. 208 patients, who were younger than 70 years and 28 patients, who had previously detected pulmonary hypertension/or left heart failure, were excluded. There was an overlap of ten patients who were younger than 70 years with existing excluding comorbidities as defined in methods. 117 patients were included in the final analysis, 58 ICU survivors and 59 non-survivors. ICU mortality was 50.9%. The general description of patients, previous history and chronic therapy is presented in Table 1. Tests for normal distributions did not reject the null hypothesis that variables were normally distributed. There was no difference in average age between survivors and non-survivors (76 ± 5 vs. 77 ± 5 years, *p* = 0.2). ICU length of stay was shorter in ICU survivors compared to non-survivors (8.8 ± 8.3 vs. 12.6 ± 9.3 days, *p* = 0.02).

**Table 1:** General description of patients, previous history and chronic therapy

Variable	All (*n* = 117)	ICU survivors (*n* = 58)	ICU non-survivors (*n* = 59)	Statistics (p)
Age, years	77 ± 5	76 ± 5	77 ± 5	0.2
Gender, female/male, n	31 / 86	22 / 36	9 / 50	0.2
Height, cm	173 ± 7	173 ± 7	173 ± 8	0.6
Body weight, kg	87 ± 16	91 ± 18	84 ± 14	0.023^a^
COVID-19 symptoms^b^, days	7 ± 4	7 ± 4	7 ± 4	0.9
ICU LOS, days	10.7 ± 9.0	8.8 ± 8.3	12.6 ± 9.3	0.02^a^
Hospital LOS, days	19.5 ± 10.8	21.3 ± 10.5	17.9 ± 10.9	0.09
**Previous history:**				
LV hypertrophy, n (%)	21 (18)	13 (22)	8 (14)	0.3
Malignant disease, n (%)	23 (20)	13 (22)	10 (17)	0.6
Arterial hypertension, n (%)	81 (70)	40 (70)	41 (70)	0.9
Diabetes, n (%)	42 (36)	21 (36)	21 (36)	0.9
COPD, n (%)	13 (11)	6 (11)	7 (12)	0.5
Chronic kidney failure, n (%)	22 (19)	10 (17)	12 (20)	0.8
**Therapy at home:**				
Statins, n (%)	37 (32)	20 (35)	17 (29)	0.3
Beta-blocker, n (%)	47 (40)	24 (42)	23 (39)	0.4
Inhalation corticosteroids, n (%)	16 (14)	8 (14)	8 (14)	0.6
ACE inhibitors, n (%)	48 (41)	23 (40)	25 (42)	0.5
Insulin, n (%)	13 (11)	6 (11)	7 (12)	0.5
Aspirin, n (%)	34 (29)	18 (32)	16 (32)	0.4
Diuretics, n (%)	25 (22)	13 (23)	12 (20)	0.5

Values represent means with standard deviations or number of subjects with percentages

*Abbreviations*: *ACE* Angiotensin-converting enzyme, *COPD* Chronic obstructive pulmonary disease, *COVID-19* Coronavirus disease 2019, *ICU* intensive care unit, *LOS* Length of stay, *SD* standard deviation, *LV* Left ventricle

^a^Denotes statistically significant difference between groups at < 0.05 level

^b^Days of COVID-19 symptoms before ICU admission

At admission there was no difference between survivors and non-survivors in vital parameters, however serum lactic values were lower in survivors (2.1 ± 1.6 vs. 3.8 ± 4.2 mmol/l, *p* = 0.004) (Table 2). Admission PaO_2_/FiO_2_ was 80.5 ± 52.1 mmHg, without difference between groups (Table 2).

**Table 2:** Clinical parameters on admission and laboratory findings during treatment

Variable	All (*n* = 117)	ICU survivors (*n* = 58)	ICU non-survivors (*n* = 59)	Statistics (p)
**At admission**				
Heart rate, bpm	90 ± 24	88 ± 24	92 ± 24	0.1
Respiratory rate, rpm	30 ± 8	28 ± 6	31 ± 9	0.3
SAP, mmHg	140 ± 29	144 ± 29	136 ± 28	0.2
DAP, mmHg	68 ± 15	68 ± 16	68 ± 15	0.8
PaO_2_/FiO_2_, mmHg	80.5 ± 52.1	89.2 ± 59.4	79.9 ± 48.1	**0.3**
Lactate, mmol/L	3.0 ± 3.3	2.1 ± 1.6	3.8 ± 4.2	0.004^b^
**Extreme values in ICU**				
Highest FiO_2_, %	93 ± 18	91 ± 21	95 ± 14	0.024^b^
Lowest pH, value	7.26 ± 0.14	7.34 ± 0.09	7.18 ± 0.13	0.001^b^
Lowest PaO_2_, kPa^c^	8.02 ± 3.43	8.29 ± 1.90	7.74 ± 4.44	0.4
Highest PaCO_2_, kPa^c^	8.43 ± 3.48	6.70 ± 2.43	10.09 ± 3.54	0.001^b^
Highest HCO_3_, mmol/L	28.9 ± 6.7	28.9 ± 6.07	30.2 ± 7.13	0.3
Lowest StHbO_2_, %	84.4 ± 8.4	86.9 ± 5.8	78.9 ± 8.6	0.001^b^
Highest creatinine, µmol/L	221 ± 195	175 ± 174	266 ± 205	0.012^b^
Highest proBNP, pg/ml^a^	12,599 ± 12,160 (*n* = 18)	6500 ± 6363 (*n* = 9)	18,693 ± 13,790 (*n* = 9)	0.028^b^
Highest troponin I, ng/ml^a^	436 ± 1518 (*n* = 86)	588 ± 2071 (*n* = 43)	284 ± 571 (*n* = 43)	0.4
Highest D-dimer, µg/L^a^	9898 ± 10,081 (*n* = 100)	6665 ± 7961 (*n* = 48)	12,881 ± 10,954 (*n* = 52)	0.002^b^
Highest PCT, ng/L	7.14 ± 17.2	5.45 ± 16.40	8.72 ± 17.91	0.3
Highest CRP, mg/L	212 ± 122	174 ± 105	248 ± 127	0.001^b^
Highest WBC count, 10^9^/L	22.09 ± 21.33	17.1 ± 17.0	26.8 ± 23.9	0.015^b^
Highest PLT count, 10^9^/L	271 ± 169	281 ± 170	262 ± 169	0.6
Highest AST, µkat/L^d^	6.33 ± 19.48	4.43 ± 18.38	8.13 ± 20.47	0.3
Highest ALT, µkat/L^d^	3.27 ± 8.18	2.34 ± 6.26	4.14 ± 9.63	0.2
Highest GGT, µkat/L^e^	3.27 ± 3.67	2.74 ± 2.34	4.19 ± 4.50	0.034^b^
Highest bilirubin, µmol/L	11.5 ± 7.19	12.5 ± 7.1	13.7 ± 7.2	0.4

Values represent means with standard deviations

*Abbreviations*: *ALT* Alanine transaminase, *AST* Aspartate aminotransferase, *BNP* Brain natriuretic peptide, *CRP* C-reactive protein, *DAP* Diastolic arterial pressur, *FiO*_*2*_ Fraction of inspired oxygen, *GGT* Gamma-glutamyl transferase, *ICU* Intensive care unit, *PaCO*_*2*_ Partial arterial carbon dioxide pressure, *PaO*_*2*_ Partial arterial oxygen pressure, *PCT* Procalcitonin, *PLT* Platelets, *SAP* Systolic arterial pressure, *StHbO*_*2*_ Oxygen saturation, *WBC* White blood cells

^a^Number in brackets represents the number of patients who had that measurement done

^b^Denotes statistically significant difference between groups at < 0.05 level

^c^Coversion factor (CF): 1 kPa = 7.5 mmHg

^d^CF: 1 µkat/L = 60.24 unit/L

^e^CF: 1 µkat/L = 59.99 unit/L

During ICU stay lower values of pH, higher pCO_2_, lower hemoglobin oxygen saturation (StHbO_2_), higher leukocyte count, higher creatinine levels, higher C-reactive protein and higher D-dimer were detected in non-survivors compared to survivors (Table 2).

All patients were treated with methylprednisolone (see Methods, Treatment subsection) (Table 3). Survivors had been shortly non-invasively ventilated (NIV) (1.5 ± 0.8 vs. 3.2 ± 2.5 days, *p* = 0.01) and were less often invasively mechanically ventilated (47% vs 81%, *p* = 0.001). There was no difference between groups in maximal PEEP or tidal volume recorded during ICU stay; however, recorded maximal peak pressure on ventilators was higher in non-survivors. Ventilator associated pneumonia (VAP) was more often detected in non-survivors (49% vs. 21%, *p* = 0.001). Comparison of VAP incidence in groups of survivors and non-survivors, who were mechanically ventilated, showed no difference (44% vs. 42%, *p* = 0.54).

**Table 3:** Respiratory support, specific treatment modalities and complications

Variable	All (*n* = 117)	ICU survivors (*n* = 58)	ICU non-survivors (*n* = 59)	Statistics (p)
**Ventilatory support**				
Self-proning, n (%)	3 (3)	1 (2)	2 (3)	1.0
High-flow, n (%)	36 (31)	15 (26)	21 (36)	0.3
Duration of high-flow, days	2.3 ± 1.6	2.3 ± 2.1	2.2 ± 1.2	0.9
NIV, n (%)	39 (34)	17 (29)	22 (37)	0.5
Duration of NIV, days	2.5 ± 2.1	1.5 ± 0.8	3.2 ± 2.5	0.01^a^
IMV, n (%)	75 (65)	27 (47)	48 (81)	0.001^a^
Duration of IMV, days	10.3 ± 8.7	8.0 ± 8.5	11.5 ± 8.6	0.09
Proning during IMV, n (%)	12 (10)	2 (3)	10 (17)	0.04^a^
Maximal PEEP, cmH_2_O	12 ± 4	11 ± 4	12 ± 3	0.2
Tidal volume, ml	536 ± 110	560 ± 93	525 ± 117	0.3
Highest Peak pressure, cmH_2_O	36 ± 7	32 ± 7	37 ± 6	0.01^a^
**Medical and renal support**				
Methylprednisolone, n (%)	117 (100)	58 (100)	59 (100)	1,0
Levosimendan, n (%)	8 (7)	5 (9)	3 (5)	0.4
Nitric oxide inhalation, n (%)	2 (2)	2 (4)	0	0.3
Thrombolysis (rTPA), n (%)	3 (3)	1 (2)	2 (4)	0.5
Renal replacement therapy, n (%)	18 (16)	4 (7)	14 (24)	0.012^a^
**Complication rate:**				
VAP, n (%)	41 (35)	12 (21)	29 (49)	0.001
CRBSI, n (%)	1 (1)	0	1 (2)	0.5
Urosepsis, n (%)	24 (21)	12 (21)	12 (10)	0.6
Fungal infection, n (%)	35 (29)	13 (22)	21 (36)	0.09

Values represent means with standard deviations or number of subjects with percentages

*Abbreviations*: *CRBSI* Catheter-related bloodstream infection, *ICU* Intensive care unit, *IMV* Invasive mechanical ventilation, *NIV* Non-invasive mechanical ventilation, *PEEP* Positive end-expiratory pressure, *rTPA* Recombinant tissue plasminogen activator, *VAP* Ventilator associated pneumonia

^a^Denotes statistically significant difference between groups at < 0.05 level

An echocardiogram was performed on all patients at admission into ICU (Table 4). There were no differences between groups in LV EF, velocity time integral in left ventricular outflow tract in systole, TAPSE or inferior vena cava diameter during respiration. PASP at admission was lower in survivors compared to non-survivors (32.5 ± 9.8 vs. 40.4 ± 14.3 mmHg, *p* = 0.024).

**Table 4:** Point-of-care heart and lung ultrasound data

Variable	All (*n* = 117)	ICU survivors (*n* = 58)	ICU non-survivors (*n* = 59)	Statistics (p)
**Heart:**				
LVEF, %	51 ± 15	50 ± 15	52 ± 14	0.5
LVOT VTI	18 ± 5	18 ± 5	19 ± 5	0.2
PASP, mmHg	36.7 ± 12.9	32.5 ± 9.8	40.4 ± 14.3	0.024^a^
TAPSE, cm	1.95 ± 0.52	1.93 ± 0.56	1.97 ± 0.49	0.7
TAPSE/PASP, cm/mmHg	0.05 ± 0.02	0.05 ± 0.02	0.05 ± 0.02	0.8
VCI min. diameter, cm	1.6 ± 0.8	1.7 ± 0.7	1.5 ± 0.8	0.3
VCI max. diameter, cm	2.1 ± 0.5	2.2 ± 0.5	2.0 ± 0.6	0.3
**Lung:**				
Diffuse B-lines pattern, n (%)	54 (46)	19 (33)	35 (59)	0.005^a^z
Mixed A-/B-lines pattern, n (%)	41 (35)	24 (41)	17 (29)	0.3
A-lines pattern, n (%)	3 (3)	3 (5)	0	0.2
Pleural effusion, n (%)	16 (13)	9 (16)	7 (12)	0.8
Lung consolidations, n (%)	3 (3)	3 (5)	0	0.2

Values represent means with standard deviations or number of subjects with percentages

*Abbreviations*: *ICU* Intensive care unit, *LVEF* Left ventricular ejection fraction, *LVOT VTI* Left ventricular outflow tract velocity time integral, *PASP* Pulmonary artery systolic pressure, *TAPSE* Tricuspid annular plane systolic excursion, *VCI* Vena cava inferior

^a^Denotes statistically significant difference between groups at < 0.05 level

At ICU admission diffuse B-line pattern and mixed B-line/A-line pattern were most often detected on lung ultrasound examination (Table 4). Diffuse B-line pattern was more often recorded in non-survivors than survivors (59% vs. 33%, *p* = 0.005).

PASP and diffuse B-line pattern were univariate predictors of ICU and hospital mortality (Table 5). PASP was the only independent predictor of ICU mortality (odds ratio [OR] 1.061, 95% confidence interval [CI] 1.003–1.124, *p* = 0.039) and also hospital (OR 1.073, 95% CI 1.003–1.146, *p* = 0.039) mortality. Results of receiver operating characteristic (ROC) curve analysis of PASP as ICU and hospital mortality predictor are presented in Tables 6 and 7.

**Table 5:** Univariate and multivariate logistic regression models

Variable in the model	OR	95% CI	Statistics (p)
**Univariate predictors of ICU mortality**			
PASP, mmHg	1.056	1.005–1.111	0.033^a^
Diffuse B-pattern (Yes = 1, No = 0)	2.917	1.368–6.218	0.006^a^
Mechanical ventilation (Yes = 1, No = 0)	4.849	2.101–11.191	< 0.001^a^
Development of VAP (Yes = 1, No = 0)	3.625	1.603–8.197	0.002^a^
**Multivariate regression model of ICU mortality** (Full model -2 Log Likelihood = 63.0, Chi-square *p* < 0.021)			
PASP, mmHg	1.061	1.003–1.124	0.039^a^
Diffuse B-pattern (Yes = 1, No = 0)	3.317	0.918–1.988	0.067
Mechanical ventilation (Yes = 1, No = 0)	1.853	0.432–7.954	0.407
Development of VAP (Yes = 1, No = 0)	2.075	0.442–9.745	0.355
**Univariate predictors of hospital mortality**			
PASP, mmHg	1.068	1.007–1.133	0.028^a^
Diffuse B-pattern (Yes = 1, No = 0)	2.230	1.000–4.975	0.050^a^
Mechanical ventilation (Yes = 1, No = 0)	5.018	2.065–12.194	< 0.001^a^
Development of VAP (Yes = 1, No = 0)	5.961	2.188–16.241	< 0.001^a^
**Multivariate regression model of hospital mortality** (Full model -2 Log Likelihood = 51.8, Chi-square *p* < 0.013)			
PASP, mmHg	1.073	1.003–1.146	0.039^a^
Diffuse B-pattern (Yes = 1, No = 0)	2.602	0.635–10.668	0.184
Mechanical ventilation (Yes = 1, No = 0)	1.275	0.282–5.755	0.752
Development of VAP (Yes = 1, No = 0)	4.786	0.729–31.436	0.103

Values represent odds ratios with 95% confidence intervals

*Abbreviations*: *CI* Confidence interval, *ICU* Intensive care unit, *OR* Odds ratio, *PASP* Pulmonary artery systolic pressure, *VAP* Ventilator associated pneumonia

^a^Denotes statistically significant difference between groups at < 0.05 level

**Table 6:** ROC curve analysis of PAPS as ICU and hospital mortality predictor

	**AUC**	**95% CI**	**Statistics (p)**
ICU	0.660	0.518–0.783	0.031^a^
Hospital	0.684	0.534–0.811	0.016^a^

*Abbreviations*: *AUC* Area under the curve, *CI* Confidence interval, *ICU* Intensive care unit, *PASP* Pulmonary artery systolic pressure, *ROC* Receiver operating characteristic

^a^Denotes statistical significance at < 0.05 level

**Table 7:** Analysis of PASP cut-off values for predicting ICU and hospital mortality as determined using ROC curves

	**Cut-off**	**Sensitivity**^**1**^	**Specificity**^**1**^	** + LR**^**2**^	**-LR**^**2**^
ICU	> 42	41.38 (23.5–61.1)	88.00 (68.8–97.5)	3.45 (1.1–10.9)	0.67 (0.5–0.9)
Hospital	> 42	41.38 (23.5–61.1)	89.47 (66.9–98.7)	3.93 (1.0–15.6)	0.66 (0.5–0.9)

*Abbreviations*: *CI* Confidence interval, *ICU* Intensive care unit, + *LR* Positive likelihood ratio, *-LR* Negative likelihood ratio; PASP, pulmonary artery systolic pressure; ROC, receiver operating characteristic

^1^Values represent percentages with 95% CI

^2^LRs represented with 95% CI

Development of VAP and mechanical ventilation were strong univariate predictors of ICU and hospital mortality, but did not retain statistical significance in multivariate models (Table 5).

## Discussion

Our study confirmed that PASP at admission is an independent predictor of ICU and also hospital mortality of elderly patients with severe COVID-19 pneumonia.

Right heart failure (“acute cor pulmonale”) is a long-recognized complication of acute respiratory distress syndrome (ARDS) in relation to severity of the disease and ventilatory strategies associated with hyper-inflated lungs and permissive hypercapnia [16, 17]. Right heart is also vulnerable in the setting of COVID-19 pneumonia [18]. In our study we have analyzed robust and easily obtainable echocardiographic parameters which we measured in all patients at admission to our ICU. We always estimated relative dimension of RV compared to left ventricle (LV) (i.e., smaller, same size, larger); we measured PASP, TAPSE and VCI respiratory dynamics. That is why we have not used more sophisticated parameters of RV function such as RV longitudinal strain obtained by speckle-tracking echocardiography.

In previous study of younger patients (age 62 ± 13 years) RV dysfunction was found in 25.5% of unselected patients, 28.9% patients requiring high-flow oxygen and 41.7% patients requiring mechanical ventilation [19]. Incidence of LV and RV dysfunction was higher in non-survivors than survivors. In multivariate Cox analysis, high-sensitivity troponin (hs-TNI) elevation, mechanical ventilation and RV dysfunction were independent predictors of higher mortality.

In small cohort of critically ill (age 64.5 ± 10 years) elevated PASP and decreased TAPSE were associated with disease severity and composite endpoints, as well as in-hospital mortality (50%). Equivalent level of mortality was also observed in the present study [18].

In 510 non-ICU patients (age 64 ± 14 years, 66% male) RV dilation and dysfunction were present in 35% and 15%, respectively [20]. Pulmonary artery pressure was higher in patients who had RV dilatation. There was no difference in TAPSE between patients with or without RV dysfunction or RV dilatation, however TAPSE values were low (1.9 ± 0.5 cm). We have also found no difference in TAPSE between survivors and non-survivors, which was low in both our groups (Table 4).

In recent systematic review and meta-analysis TAPSE was related to mortality in unselected COVID-19 patients [21]. Every 1 mm decrease in TAPSE was associated with an increase in mortality of approximately 20%.

In study analyzing predictive role of combined cardiac and lung ultrasound in 200 COVID-19 non-ICU patients (age 64.2 ± 19.2 years) hemodynamic right-side assessment included measurement of pulmonic flow acceleration time velocity to assess pulmonary vascular resistance, surrogate of pulmonary artery hypertension, and estimated right atrial pressure using the inferior vena cava [22]. Estimation of systolic pulmonary pressure on the basis of tricuspid regurgitation pressure gradient was possible in only 18%, compared to 100% in our older critically ill. In this study the only echocardiographic parameters associated with adverse outcome in non-adjusted analyses were LVEF, stroke volume index, pulmonic flow acceleration time, and TAPSE. The cutoff values for TAPSE and LVEF were within the lower normal range and thus unlikely to be discriminatory in other populations [22]. However, because of the heightened adrenergic tone in patients with respiratory failure, a ‘‘lower normal range’’ TAPSE or LVEF may reflect early cardiac deterioration. As mentioned previously, in our study, TAPSE, which was low in both groups, was not discriminatory.

More advanced echocardiography based on speckle-tracking (e.g., global LV strain, RV longitudinal strain), which can detect subtle function changes [23], were also studied in COVID-19 patients. In 120 non-ICU patients (age 61 ± 14 years) LV and RV strain was abnormal in 40% of patients [24]. Poorer clinical grade and clinical deterioration were mostly associated with worsening RV segmental strain, in a pattern suggestive of acute cor pulmonale. LV and RV strains were strong predictors of mortality and need for intubation in patients with SARS-CoV-2 infection.

Pulmonary artery hypertension in the present study was mild to moderate as based on echocardiographic estimates of PASP. A mean PASP of 40 mmHg in the non-survivors would indeed be at the upper limit of normal range, taken into account age, sex, and body weight [25]. On the other hand, TAPSE was decreased but still above the lower limit of normal (18 mm) in non-survivors.

In a recently published study (age of survivors (*n* = 69) 62 ± 13 years vs age of non-survivors (n = 25) 68 ± 12 years), authors hypothesized that myocardial injury and inflammatory changes in COVID-19 could be additional causes of ARDS-related acute right heart failure [26]. They therefore assessed the coupling of RV function to the pulmonary circulation in COVID-19 ARDS patients. For this purpose, they used bedside transthoracic echocardiography with focus on TAPSE/PASP ratio, previously shown to be a valid surrogate of the gold standard ratio of end-systolic to arterial elastance (Ees/Ea) for the assessment of RV-arterial coupling [27] and an independent predictor of outcome in heart failure and pulmonary arterial hypertension [28].

TAPSE/PASP is easier to assess and can therefore be part of standard bedside echocardiographic assessments as it does not require off-line analysis of images, specific software and may be a more sensitive assessment of RV-pulmonary artery coupling. High prevalence of RV dilatation and dysfunction in the range of 40–50% recently reported in patients with COVID-19 underscore the exquisite sensitivity of the RV to this newly appeared viral infection [29, 30]. In their study TAPSE/ PASP emerged with equally potent prognostic capability for mortality (HR 0.026, 95% CI 0.01–0.58, *p* = 0.019), suggesting a major component of acute cor-pulmonale in COVID-19 ARDS pathophysiology. ROC-determined cut-off TAPSE/PASP value of 0.635 mm/mmHg. In our study survivors and non-survivors had low TAPSE/PASP values compared to upper mentioned cut-off. In our study there was no difference in TAPSE/PASP ratio between groups, this could be related to age and severity of the disease.

Previous study not focused on elderly confirmed that markers of RV (TAPSE < 18.5 mm) and LV (LVEF < 64%) dysfunction assessed by bedside echo and older age (age ≥ 63 years) were independent predictors of mortality in hospitalized moderate to severely ill COVID-19 patients [31]. In this study other variables of RV function, such as fractional area change, were higher, and also RV basal dimension was shorter in survivors. Surprisingly, authors have not detected any difference in PASP between survivors and non-survivors.

As in COVID-19 ARDS patients, the relationship of PASP and TAPSE was investigated on cohorts of all-cause ARDS patients. Investigators of European Collaborative ARDS Study [32] determined elevated PASP on admission (and at 48 h) as independent predictor of mortality as consistent with our COVID-19 cohort. Lower TAPSE values were strongly predictive of higher mortality in cohort of 38 patients [33], in contrast to our study in which we did not find relationship between TAPSE and mortality.

The pathophysiology of RV is complex and multifactorial. Direct viral damage, aggravation of systemic inflammatory response and hypoxemia may all contribute to cardiac injury [34]. Furthermore, RV function can be worsened by increased afterload, which is likely to be caused by ARDS, hypoxic pulmonary vasoconstriction, micro-thrombi within the pulmonary vasculature and microvascular injury. More research is needed to elucidate the inflammatory pathways and myocardial pathology responsible for RV dysfunction in patients with COVID-19, and determine whether survivors with pathological RV remodeling remain at risk of adverse outcomes [20].

The most potent predictor of outcome in ARDS is the PaO_2_/FiO_2_ ratio, which as such is part of the definition of the syndrome [35]. The PaO_2_/FiO_2_ ratio was severely low in our cohort. Most patients in our study have pronounced changes detected on LUS, almost half of included patients and almost 60% of non-survivors had diffuse B-pattern. B-pattern was univariate predictor of ICU and hospital mortality; however, it lost its predictive power in model with PASP, VAP development and mechanical ventilation. Use of LUS as a diagnostic tool in critically ill patients for establishing the degree of parenchymal involvement, to assess treatment response, and during follow-up is a common practice that has become a high-quality patient bedside standard of care [36].

Mortality of our cohort was in range of one other study with the same age range of included patients [37]. Ventilator associated pneumonia in our patients was a complication, which was associated with increased ICU mortality. Incidence of VAP in our older cohort was higher compered to previously published data (age 64 years, IQR 57–71 years), 35% vs. 29%, respectively; however, the fatality rates were identical [13]. In previous study septic shock at VAP onset (OR 3.30, 95% CI 1.43–7.61, *p* = 0.005) and acute respiratory distress syndrome at VAP onset (OR 13.21, 95% CI 3.05–57.26, *p* < 0.001) were strongly associated with mortality [13].

Recent documents published by the European Association of Cardiovascular Imaging and the American Society of Echocardiography have recommended a FoCUS approach in patients with COVID-19 [38, 39]. As these guidelines were based on expert opinion rather than outcome data, we aimed to assess whether an even more limited approach is sufficient. We found that an optimal model including only one echocardiographic parameter, PASP, provides information that is potentially valuable for clinical management of elder critically ill with severe COVID-19 pneumonia without previously known pulmonary artery hypertension and with preserved LV function. Further prospective study using easy obtainable parameters confirming our data would be appreciated.

## Study limitations

Our study has at least four weaknesses. The first major limitation is, that this is a retrospective cohort study. Therefore, we have only been able to collect and re-evaluate everyday ICU clinical practice echocardiography and LUS data. On the other hand, we perceive this also as an advantage, especially because both ultrasound examinations are part of local standard at admission protocol in our ICU for all patients, COVID and non-COVID. Second major limitation is that lung ultrasound was performed only on anterior and lateral regions, omitting posterior lung segments. We acknowledge that this approach could decrease predictive power of LUS examination and are aware that it would be even more useful to also inspect posterior lung areas. We omitted that in light of practical difficulty. Most our patients were unable to actively participate when examined. For posterior examination sitting position is required which would necessitate additional personnel, which was in especially short supply during COVID-19 pandemic. Time and personnel limitations were, unfortunately, main limiting factors that we did not revise our approach to perform more advanced lung ultrasound examination at admission, e.g., 12-zone lung ultrasound protocol [40]. We still perceive our approach as a positive, especially in light of the fact that our approach resembles similarities to the one of Levy Adatto et al. [9] who compared rapid 8-zone lung ultrasound protocol to a full 12-zone protocol for outcome prediction in hospitalized COVID-19 patients in which they also omitted posterior lung areas examination and found good correlation between the two. Thirdly, we acknowledge that more sophisticated and time-consuming echocardiographic methods (e.g., speckle-tracking) would confirm additional and subtler RV dysfunctions; however, in critically ill, heart dysfunctions are usually more prominent and that is why they can also be easily detected with robust methods. Fourth major limitation is relatively low number of included patients. Number of included patients has not allowed us to construct and test more complex regression models.

## Conclusions

Current retrospective cohort study, which included elderly critically ill patients with severe COVID-19 pneumonia without previous history of heart failure or elevated PASP, confirmed that PASP at admission predicts the ICU and the hospital mortality.

## Data Availability

The datasets used and/or analyzed during the current study are available from the corresponding author on reasonable request.

## Abbreviations

ACE: Angiotensin-converting enzyme
ALT: Alanine transaminase
ARDS: Acute respiratory distress syndrome
AST: Aspartate aminotransferase
AUC: Area under the curve
BMI: Body mass index
BNP: Brain natriuretic peptide
B-pattern: Diffuse B-line pattern
CI: Confidence interval
COPD: Chronic obstructive pulmonary disease
COVID-19: Coronavirus disease 2019
CRBSI: Catheter-related bloodstream infection
CRP: C-reactive protein
CVP: Central venous pressure
DAP: Diastolic arterial pressure
EF: Ejection fraction
FiO_2_: Fraction of inspired oxygen
FoCUS: Focused cardiac ultrasound study
GGT: Gamma-glutamyl transferase
HFNC: High-flow nasal cannula
HR: Hazard ratio
hs-TnI: High sensitivity troponin I
ICU: Intensive care unit
IMV: Invasive mechanical ventilation
LOS: Length of stay
LUS: Lung ultrasound
LV: Left ventricle
LVOT: Left ventricular outflow tract
NIV: Non-invasive mechanical ventilation
OR: Odds ratio
PASP: Pulmonary artery systolic pressure
PaCO_2_: Partial arterial carbon dioxide pressure
PCT: Pro-calcitonin
PEEP: Positive end-expiratory pressure
PaO_2_: Partial arterial oxygen pressure
POCUS: Point-of-care ultrasound
ROC: Receiver operating characteristic
rTPA: Recombinant tissue plasminogen activator
RT-PCR: Real-time polymerase chain reaction
RV: Right ventricle
SAP: Systolic arterial pressure
SARS-CoV-2: Severe acute respiratory syndrome coronavirus 2
StHbO_2_: Oxygen saturation
TAPSE: Tricuspid annular plane systolic excursion
VAP: Ventilator-associated pneumonia
VTI: Velocity time integral
VCI: Vena cava inferior
WBC: White blood cell
